# Genome-Wide Association Study of Pancreatic Cancer in Japanese Population

**DOI:** 10.1371/journal.pone.0011824

**Published:** 2010-07-29

**Authors:** Siew-Kee Low, Aya Kuchiba, Hitoshi Zembutsu, Akira Saito, Atsushi Takahashi, Michiaki Kubo, Yataro Daigo, Naoyuki Kamatani, Suenori Chiku, Hirohiko Totsuka, Sumiko Ohnami, Hiroshi Hirose, Kazuaki Shimada, Takuji Okusaka, Teruhiko Yoshida, Yusuke Nakamura, Hiromi Sakamoto

**Affiliations:** 1 Laboratory of Molecular Medicine, Human Genome Center, Institute of Medical Science, the University of Tokyo, Tokyo, Japan; 2 Department of Medical Genome Sciences, Graduate School of Frontier Sciences, the University of Tokyo, Tokyo, Japan; 3 Genetics Division, National Cancer Center Research Institute, Tokyo, Japan; 4 Laboratory for Statistical Analysis, Center for Genomic Medicine, RIKEN, Tokyo, Japan; 5 Laboratory for Genotyping Development, Center for Genomic Medicine, RIKEN, Kanagawa, Japan; 6 Statistical Genetics Analysis Division, StaGen Co., Ltd., Tokyo, Japan; 7 Science Solutions Division, Mizuho Information and Research Institute, Inc., Tokyo, Japan; 8 Bioinfomatics Group, Research and Development Center, Hitachi Government and Public Corporation System Engineering Ltd., Tokyo, Japan; 9 Department of Internal Medicine, Keio University School of Medicine, Tokyo, Japan; 10 Hepatobiliary and Pancreatic Surgery Division, National Cancer Center Hospital, Tokyo, Japan; 11 Hepatobiliary and Pancreatic Oncology Division, National Cancer Center Hospital, Tokyo, Japan; Duke University Medical Center, United States of America

## Abstract

Pancreatic cancer shows very poor prognosis and is the fifth leading cause of cancer death in Japan. Previous studies indicated some genetic factors contributing to the development and progression of pancreatic cancer; however, there are limited reports for common genetic variants to be associated with this disease, especially in the Asian population. We have conducted a genome-wide association study (GWAS) using 991 invasive pancreatic ductal adenocarcinoma cases and 5,209 controls, and identified three loci showing significant association (*P-*value<5×10^−7^) with susceptibility to pancreatic cancer. The SNPs that showed significant association carried estimated odds ratios of 1.29, 1.32, and 3.73 with 95% confidence intervals of 1.17–1.43, 1.19–1.47, and 2.24–6.21; *P*-value of 3.30×10^−7^, 3.30×10^−7^, and 4.41×10^−7^; located on chromosomes 6p25.3, 12p11.21 and 7q36.2, respectively. These associated SNPs are located within linkage disequilibrium blocks containing genes that have been implicated some roles in the oncogenesis of pancreatic cancer.

## Introduction

Pancreatic cancer is the fifth leading cause of cancer death with an estimated death of 24,634 patients in Japan in year 2007. Its 5-year survival rate is as low as 6.7% (http://www.fpcr.or.jp/publication/pdf/statistics2009/fig01.pdf and http://www.fpcr.or.jp/publication/pdf/statistics2009/fig20.pdf). Since no specific symptom is observed in the patients with pancreatic cancer at an early stage, most of the patients were diagnosed at their advanced stage with a very low possibility of cure for the disease [1], [2].

Previous reports indicated the involvement of both environmental and genetics factors in the etiology of this deleterious disease. Several case-control and cohort epidemiological studies have identified a number of possible risk factors such as smoking [3], diabetes [4], chronic pancreatitis [5], which are likely to predispose individual to the disease. In addition, familial aggregation of the disease has implied the possible involvement of genetic factors in pancreatic cancer [6]; approximately 10% of the patients were reported to have family history and individuals having first-degree relatives with pancreatic cancer revealed 2- to 4- fold higher risk of the disease [7]–[9]. These data indicated that genetic factors are likely to play some roles in the development of pancreatic cancer. In the last decade, the advancement of molecular biology improved the understanding of the pathogenesis of pancreatic cancer and characterized a number of genes that mutated in pancreatic cancers, such as somatic mutations in genes *INK4A(CDKN2A), TP53, DPC4, BRCA1/2*, *STK11, APC, KRAS* and *ATM and PALB2* are found in pancreatic cancers [10]–[18].

Two recent GWAS studies for pancreatic cancer using Caucasian populations have identified associations with genome-wide significance on chromosomes 9p34.2 (*ABO*), 13q22.1, 1q32 (*NR5A2*) and 5p15.33 (*CLPTM1L-TERT*), and highlighted that accumulation of these common genetic risk variants with modest effects are likely to play an important role on this complex disease, either individually or in interaction with environmental factors [19]–[22]. As the ethnicity is one of the critical factors in the pathogenesis of the genetic diseases with complex gene-gene and gene-environmental interactions, we (Biobank Japan (BBJ) in The University of Tokyo and National Cancer Center (NCC) Japan) combined samples of 991 cases with pancreatic cancer and 5209 controls (Table S1), attempted to identify common genetic variations associated with susceptibility to pancreatic cancer in the Japanese population.

## Results

**Figure 1:**
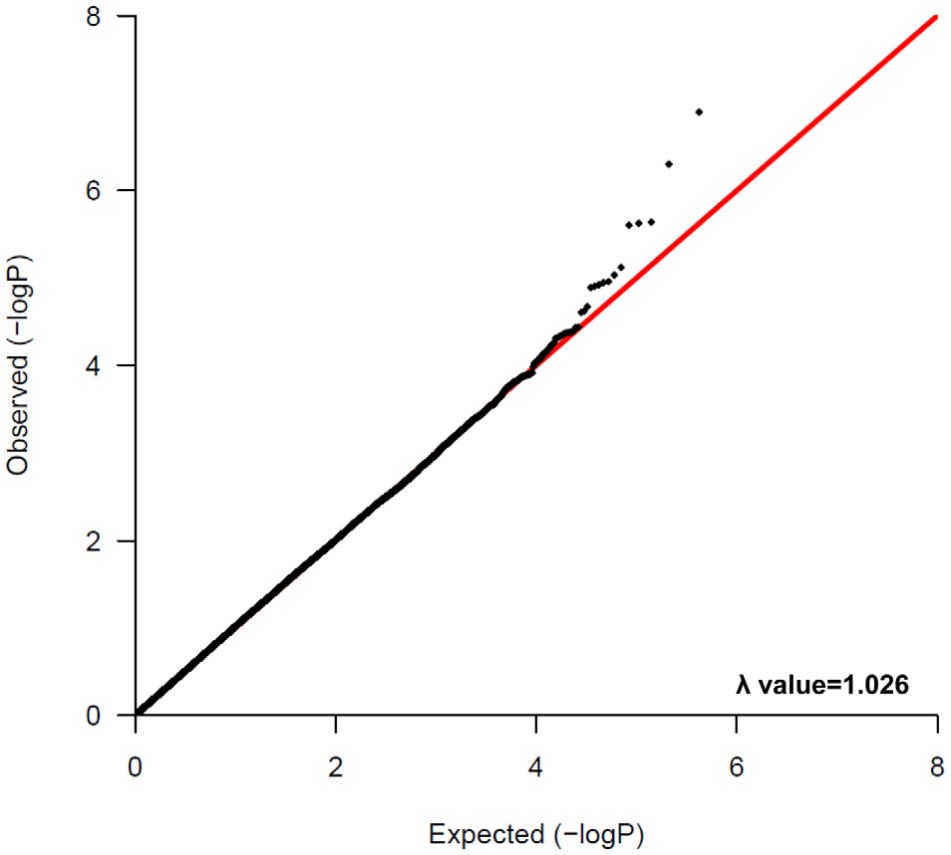
Q-Q plot for GWAS of pancreatic cancer in Japanese population. This Q-Q plot is based on logistic regression allelic *P*-values after standard quality control. (genomic inflation factor λ = 1.026).

After the standard quality control of the genotype results (Table S2), association analysis was performed for 420,236 SNPs using logistic regression analysis on the basis of allelic, dominant and recessive models after adjustment of age, sex and smoking status for each individual. The Q-Q plot for this GWAS based on allelic *P*-values by logistic regression revealed no significant population stratification with genomic inflation factor λ of 1.026 (Figure 1).

**Figure 2:**
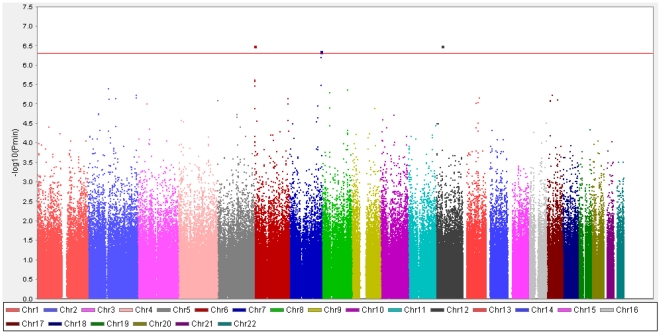
Manhattan plot for GWAS of pancreatic cancer in Japanese population. The plot is based on logistic regression model after correction of age, sex and smoking status. The *P*_min_ indicates the minimum *P*-value from logistic regression analysis for three models: allelic, dominant and recessive. Red line indicates genome-wide significant level (*P-*value = 5×10^−7^).

We successfully identified three genomic regions, 6p25.3, 12p11.21 and 7q36.2, shown to be significantly associated (*P-*value<5.0×10^−7^) with increased risk of pancreatic cancer in Japanese population as indicated in the Manhattan plot in Figure 2 (referred to ref. 23).

**Figure 3:**
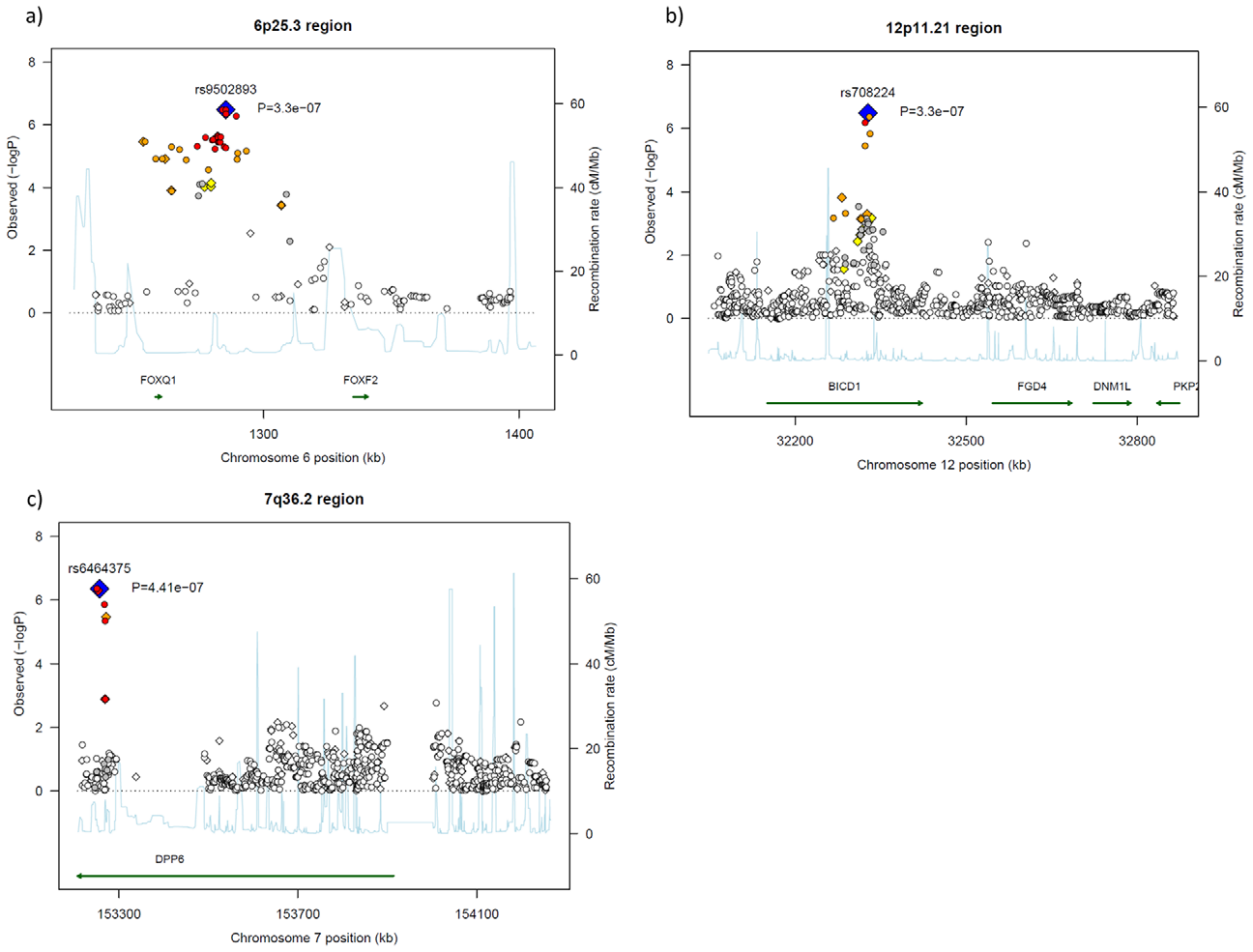
Regional association plots for three pancreatic cancer risk loci. (a) 6p25.3 region, SNP rs9502893 located 25 kb upstream to gene *FOXQ1*. (b) 12p11.21 region, SNP rs708224 is located at the second intron of gene *BICD1*. (c) 7q36.2 region, SNP rs6464375 is located at the first intron of gene *DPP6 transcript variant 3*. Each of the marker SNPs is marked by a blue diamond. SNPs that are genotyped in the Illumina platform are plotted as diamonds; Imputed SNPs are plotted as circles. The color intensity reflects the extent of LD with the marker SNP, red (r^2^≥0.8), orange (0.5≤r^2^<0.8), yellow (0.2≤r^2^<0.5) and white (r^2^<0.2). Light blue line indicated local recombination rate.

The most significantly-associated SNP, rs9502893 (*P*-value of 3.30×10^−7^, per-allele odds ratio (OR) of 1.29 with 95% confidence interval (CI) of 1.17–1.43), is located within a 75-kb linkage disequilibrium (LD) block on chromosome 6p25.3 (Table 1). This LD block includes *FOXQ1* (forkhead box (Fox) Q1) gene, which is located 25 kb upstream to this marker SNP (Figure 3a). Imputation analysis also revealed modest association at SNPs located near to or on the *FOXQ1* gene suggesting it to be one of the causative genes for pancreatic cancer (Figure 3a and Table S3).

**Table 1:** SNPs that show suggestive association with increase risk of pancreatic cancer in Japanese population.

CHR*	SNP	Position*	Risk allele	RAF		Allelic				Dominant				Recessive				*P*_min_	Gene	Relativeloc*
				Case	Control	*P-*value	OR	L95	U95	*P-*value	OR	L95	U95	*P-*value	OR	L95	U95			
6	rs9502893	1285189	G	0.411	0.351	3.30E-07	1.29	1.17	1.43	2.97E-05	1.36	1.18	1.57	2.18E-05	1.50	1.24	1.80	3.30E-07	*FOXQ1*	25196
12	rs708224	32327676	A	0.718	0.656	3.30E-07	1.32	1.19	1.47	8.54E-07	1.42	1.23	1.63	2.09E-03	1.46	1.15	1.86	3.30E-07	*BICD1*	0
7	rs6464375	153256776	A	0.116	0.103	1.15E-01	1.13	0.97	1.32	7.36E-01	1.03	0.87	1.22	4.41E-07	3.73	2.24	6.21	4.41E-07	*DPP6*	0
7	rs7779540	153253595	A	0.116	0.103	1.08E-01	1.14	0.97	1.33	7.12E-01	1.03	0.87	1.23	4.58E-07	3.72	2.23	6.20	4.58E-07	*DPP6*	0
7	rs6973850	153269181	A	0.116	0.106	2.23E-01	1.10	0.94	1.29	9.76E-01	1.00	0.84	1.18	6.27E-07	3.64	2.19	6.04	6.27E-07	*DPP6*	0
6	rs11242679	1282311	A	0.366	0.311	2.40E-06	1.28	1.15	1.42	1.15E-05	1.37	1.19	1.58	2.07E-03	1.39	1.13	1.71	2.40E-06	*FOXQ1*	22318
6	rs7750826	1281867	G	0.365	0.311	2.57E-06	1.28	1.15	1.41	1.30E-05	1.37	1.19	1.57	1.98E-03	1.39	1.13	1.71	2.57E-06	*FOXQ1*	21874
7	rs10487687	153271407	A	0.150	0.136	8.99E-02	1.13	0.98	1.30	6.76E-01	1.03	0.88	1.21	3.35E-06	2.66	1.76	4.02	3.35E-06	*DPP6*	0
6	rs11242674	1252846	A	0.355	0.301	3.46E-06	1.28	1.15	1.41	9.64E-06	1.37	1.19	1.58	4.59E-03	1.37	1.10	1.69	3.46E-06	*FOXQ1*	−4829
2	rs6711606	101288602	A	0.135	0.116	1.27E-02	1.20	1.04	1.39	1.86E-01	1.12	0.95	1.32	4.02E-06	2.81	1.81	4.37	4.02E-06	*RNF149*	0
8	rs10088262	124834883	A	0.374	0.341	3.42E-03	1.16	1.05	1.28	4.30E-06	1.40	1.21	1.61	3.98E-01	0.91	0.74	1.13	4.30E-06	*FAM91A1*	−15180
8	rs7832232	38588460	A	0.483	0.454	1.43E-02	1.13	1.03	1.25	7.63E-01	0.98	0.84	1.14	5.10E-06	1.45	1.24	1.71	5.10E-06	*RNF5P1*	−10528
2	rs6736997	235279936	A	0.372	0.328	2.95E-04	1.20	1.09	1.33	4.96E-02	1.15	1.00	1.33	5.85E-06	1.57	1.29	1.91	5.85E-06	*ARL4C*	−209504
17	rs225190	27901771	G	0.410	0.360	5.99E-06	1.26	1.14	1.39	1.92E-04	1.32	1.14	1.52	2.37E-04	1.43	1.18	1.72	5.99E-06	*MYO1D*	0
2	rs4663158	235263691	A	0.397	0.352	1.39E-04	1.21	1.10	1.34	2.89E-02	1.17	1.02	1.35	6.91E-06	1.53	1.27	1.85	6.91E-06	*ARL4C*	−193259
13	rs2039553	79197723	A	0.291	0.268	1.32E-02	1.15	1.03	1.28	4.39E-01	1.06	0.92	1.21	7.01E-06	1.73	1.36	2.19	7.01E-06	*NDFIP2*	171501
2	rs1427593	137271694	A	0.110	0.080	1.55E-05	1.42	1.21	1.66	7.10E-06	1.49	1.25	1.77	4.30E-01	1.31	0.67	2.58	7.10E-06	*THSD7B*	−193238
6	rs3016539	162156065	A	0.903	0.871	1.67E-05	1.42	1.21	1.67	7.28E-06	1.50	1.26	1.79	2.90E-01	1.36	0.77	2.43	7.28E-06	*PARK2*	0
2	rs12615966	104745389	A	0.112	0.097	1.40E-02	1.22	1.04	1.42	1.59E-01	1.13	0.95	1.35	7.44E-06	3.15	1.91	5.21	7.44E-06	*LOC284998*	−6744
17	rs2257205	53803296	A	0.378	0.327	1.58E-05	1.25	1.13	1.38	7.74E-06	1.38	1.20	1.59	2.97E-02	1.25	1.02	1.53	7.74E-06	*RNF43*	0
5	rs6879627	2162901	G	0.575	0.522	8.12E-06	1.25	1.14	1.39	4.66E-04	1.31	1.12	1.52	1.57E-04	1.42	1.18	1.69	8.12E-06	*LOC731559*	225138
17	rs4924935	18694595	G	0.269	0.228	8.80E-05	1.25	1.12	1.40	8.15E-06	1.37	1.19	1.58	5.06E-01	1.11	0.82	1.48	8.15E-06	PRPSAP2	(7622
17	rs1737947	18772157	G	0.252	0.212	3.88E-05	1.27	1.13	1.43	8.49E-06	1.37	1.19	1.58	2.89E-01	1.19	0.87	1.62	8.49E-06	PRPSAP2	0
13	rs1886449	72830115	A	0.424	0.383	2.61E-04	1.21	1.09	1.33	5.62E-02	1.15	1.00	1.33	9.24E-06	1.51	1.26	1.80	9.24E-06	*LOC730242*	−206271
13	rs1585440	65379816	C	0.761	0.713	9.28E-06	1.30	1.16	1.45	3.09E-05	1.35	1.17	1.55	7.36E-03	1.50	1.12	2.03	9.28E-06	*LOC387933*	−118820
3	rs4683235	46476935	A	0.215	0.173	9.93E-06	1.31	1.16	1.48	7.53E-05	1.34	1.16	1.54	2.24E-03	1.70	1.21	2.38	9.93E-06	*LTF*	0

Odds ratios, 95% confidence limits and P-values were obtained using logistic regression analysis according to allelic, dominant and recessive model after adjustment of age, sex and smoking.

RAF, risk allele frequency; OR, odds ratio; L95, U95, lower and upper confidence limits; *P*_min_, minimum *P*-value among three genetic models.

*Position and relative loci (Relativeloc) are based on NCBI Human Genome Build 36.

The second significantly-associated SNP, rs708224, located in the second intron of the gene *BICD1* (Bicaudal-D homolog 1) on chromosome 12p11 (*P*-value of 3.30×10^−7^, per-allele OR of 1.32 with 95% CI of 1.19–1.47) (Table 1). The 80-kb LD block showing the association corresponds to the second intron of *BICD1* as revealed by the imputation analysis shown in Figure 3b (Table S3).

The third locus is marked by rs6464375, rs7779540, rs6973850 and rs1048768 in the first intron of *DPP6* gene. These SNPs indicated suggestive associations only under recessive model with minimum *P*-value of 4.41×10^−7^ (OR of 3.73 with 95%CI of 2.24–6.21) as shown in Table 1 and Figure 3c.

## Discussion

Here we present results of GWAS analysis on 991 cases with pancreatic cancer and 5209 controls. Our study represents the first GWAS attempt to identify common variants associated with pancreatic cancer in Japanese population and successfully identified SNPs located on chromosomal loci of 6p25.3, 12p11.21 and 7q36.2 are significantly associated with increased risk of pancreatic cancer in Japanese population.

It is known that the development of the common disease is caused by the accumulation of common genetic variants, and each of this variant has a very modest effect on the risk (for example OR of <1.2). In order to detect such small fraction, GWAS involving much larger populations (5000–10000) should be required. Our study was expected to identify SNPs with moderate effects (i.e OR>1.4). Hence SNPs that show very modest effect might have failed to be identified through this study.

The most significantly associated SNP in this GWAS, rs9502893 (*P*-value = 3.30×10^−7^, OR = 1.29) is located within a 75 kb LD block which encompasses gene *FOXQ1* on chromosome loci 6p25.3. *FOXQ1* encodes for protein forkhead box (Fox) Q1. The Fox family of transcription factors consists of at least 43 members and mutations in Fox genes can cause significant effects on human common disease and cancers [24], [25]. A Fox member, FoxM1, is well-known to be associated with oncogenesis of pancreatic cancer. Down-regulation of this protein results in the inhibition of migration, invasion and angiogenesis in pancreatic cancer cells [26]. Furthermore, a recent study showed that FoxQ1 is overexpressed in pancreatic cancer, suggesting its role in pancreatic cancer tumorigenesis [27]. Although the SNP that we identified is approximately 25 kb downstream to this gene, the associated SNP may ‘tag’ the causative variant located on the expression regulatory region of the gene and subsequently alter expression of the gene. However, further study is needed to elucidate a precise biological role and mechanism of the gene function with regard to pancreatic carcinogenesis.

The second most significantly associated SNP, rs708224 (*P*-value = 3.30×10^−7^, OR = 1.32) is located within the *BICD1* gene. This gene encodes a protein Bicaudal-D homolog 1, which plays a role in vacuolar trafficking. Previous studies reported substantial evidences indicating a link between vacuolar gene and shorter telomeres in yeast model [28]–[30]. In addition, Mangino et al. suggested that genetic variations within the *BICD1* gene could alter its transcriptional levels and in turn influence telomere length in humans [31]. Several recent studies have documented reduced telomere length in pancreatic ductal adenocarnoma specimens, suggesting telomeric dysfunction in pancreatic cancer cells [32]–[34]. Thus, it is of importance to determine the functional consequences of rs708224 and/or variations linked to this SNP in the pathogenesis of pancreatic cancer.

Several SNPs located in the first intron of *DPP6* indicated suggestive associations with an increased risk of pancreatic cancer in this study. *DPP6* encodes protein dipeptidyl-peptidase 6, which binds to specific voltage-gated potassium channels and alters their expression and biophysical properties. A recent study on core signaling pathways in human pancreatic cancers found three somatic mutations in DPP6 among 24 pancreatic cancer samples examined by detailed sequence analyses. This report also suggested that DPP6 might play a crucial role in regulation of invasion of pancreatic cancer cells [35]. Hence, our study strengthens the risk of DPP6 in pancreatic cancer and warrants further screening on this gene to confirm its association with pancreatic cancer.

Recent GWAS reports have indicated several loci on chromosomes 9p34.2, 13q22.1, 1q32.1 and 5p15.33 to be associated with an increased risk of pancreatic cancer in Caucasian population [21], [22]. Among the significantly associated SNPs, rs9543325 on chromosome 13q22.1 showed moderate association in our study populations (*P*-value (allelic model) of 1.69×10^−4^; OR of 1.21 with 95%CI of 1.10–1.34) (Table S4). On the other hand, SNPs on chromosomes 9p34.2 (rs505922) and 1q32.1 (rs3790844) showed a weak association in our study populations (*P*-values of 3.69×10^−2^ and 1.24×10^−2^; ORs of 1.11 and 1.14 with 95% CI of 1.01–1.22 and 1.03–1.27, respectively) (Table S4). We were unable to replicate the remaining loci (*SHH* and two loci on chromosomes 5p15.33 and 15q14) in these reports, probably because most of these associated SNPs are either non-polymorphic or possess very low allelic frequencies (MAF = 0.01) in Japanese population. The power of our study was not sufficient enough to detect positive associations for these variants with the low allelic frequency. Such ethnic difference in genetic architecture of disease susceptibility is not rare. For example, two recent GWAS reported common variants on *KCNQ1* gene associated with type 2 diabetes mellitus in Japanese population, but European GWAS were unable to identify the associations due to the low allelic frequency of these variants in the population [36], [37]. In addition, identification of susceptibility loci may be also influenced by the differences in the LD structure across different populations and by potential interaction with other genetic variants and environmental factors [38].

In summary, this study represents the first GWAS to identify common variants possibly associated with pancreatic cancer in Japanese population. Our study confirmed the association from the Caucasian GWAS studies and revealed several novel possible candidate associated loci that were not detected in the previous Caucasian GWAS studies. Nevertheless, further additional replications are required to confirm or exclude the current findings.

## Materials and Methods

### Case and control subjects

A total of 331 and 675 cases that were clinically and/or histologically diagnosed to have an invasive pancreatic ductal adenocarcinoma were obtained from Biobank Japan (http://biobankjp.org) at the Institute of Medical Science, The University of Tokyo as well as National Cancer Center Hospital, respectively. The control samples consisted of Japanese volunteers that were obtained from Osaka-Midosuji Rotary Club, Osaka, Japan (*n* = 906) as well as from staff members in Keio University, Japan, who participated in its health-check program (*n* = 677). In addition, individuals who were registered in Biobank Japan as subjects with various diseases except cancer (*n* = 3,728) (those having pulmonary tuberculosis, chronic hepatitis-B, keroid, drug-induced skin rash, peripheral artery disease, arrhythmia, stroke and myocardial infarction) were used as controls. All samples were obtained after obtaining the written informed consent. This project was approved by the ethics committee at The Institute of Medical Sciences, The University of Tokyo, National Cancer Center and Keio University. Individuals who had clinical history of diabetes mellitus (a possible confounding factor for pancreatic cancer) were excluded from these control sets. For sample quality control, we excluded five cases with call rate<0.98. After performing principal component analysis, we excluded outliers of 10 cases and 102 controls, who did not belong to the major Japanese cluster (Hondo cluster) (Figure S1) [39]. We eventually performed the association study based on 991 cases and 5209 controls (Table S1). Power calculation showed that our study would have over 90% power to detect a per-allele OR of 1.4 or greater for an allele with 30% frequency at the genome-wide significance level (α = 5×10^−7^).

### SNP genotyping and quality control

All the individuals were genotyped using either Illumina Infinium HumanHap550v3 or Illumina Infinium Human610-Quad DNA Analysis Genotyping BeadChip. SNPs common in the two platforms were used for further analysis. We applied SNP quality control for all sets of samples as follows; SNP call rate should be >0.99 in both cases and controls, and *P*-value of Hardy-Weinberg equilibrium test should be >1.0×10^−6^ in controls. SNPs with minor allele frequency (MAF) of <0.01 in both case and control samples were excluded from the further analysis (Table S2).

### Statistical analysis

We analyzed each SNP using logistic regression adjusted for age (continuous), sex and smoking status (current/former, never). *P-*values and OR with 95%CI were calculated for allelic, dominant and recessive models. We used the minimum *P*-values obtained from three models to evaluate the statistical significance of the association. All OR were reported with respect to the risk allele. All the statistical analyses were performed using R statistical environment version 2.9.0 (http://www.r-project.org/) or PLINK 1.06 (http://pngu.mgh.harvard.edu/~purcell/plink/). R statistical environment version 2.9.0 was employed to draw Q-Q plot and regional association plot.

### Genotype Imputation

We performed genotype imputation analysis for each set of samples by utilizing a Hidden Markov model as programmed in MACH version 1.0 (http://www.sph.umich.edu/csg/abecasis/mach/index.html). To infer untyped and missing genotypes around the candidate chromosomal loci, we provided genotypes from our own samples together with haplotypes for reference samples (Japanese from Tokyo, JPT) from HapMap database (http://hapmap.ncbi.nlm.nih.gov/). SNPs with low genotyping rate (<99%), showing deviations from Hardy-Weinberg equilibrium (<1.0×10^−6^), or MAF (<0.01) were excluded from the analysis. MACH version 1.0 was used to estimate haplotypes, map crossover and error rates using 50 iterations of the Markov chain Monte Carlo algorithm. By utilizing the genotype information from the HapMap database, maximum likelihood genotypes were generated. For quality control, we retained imputed SNPs with the estimated r^2^ of >0.3. We also picked up a total of 17 SNPs (*P*-value<0.001) to verify the association using Invader and TaqMan genotyping methods (data not shown).

## Supporting Information

Table S1 Sample characteristic of this study.
(0.02 MB XLS)

Table S2 Total number of SNPs excluded according to each quality control criteria.
(0.02 MB XLS)

Table S3 Imputation analysis around significantly associated SNPs.
(0.04 MB XLS)

Table S4 Association study of SNPs which shown to be significantly associated with increased risk of pancreatic cancer in Caucasian population in Japanese.
(0.02 MB XLS)

Figure S1 Principal component analysis for GWAS of pancreatic cancer in Japanese population. a) Principal component analysis for GWAS of pancreatic cancer in Japanese population refer to four HapMap population control subjects including CEU indicates Caucasians from Utah; YRI, Nigerians from Yoruba; CHB, Han Chinese from Beijing and JPT, Japanese from Tokyo. b) Principal component analysis of study subjects referred only to Asian populations. We utilized samples from the homogenous case-control (Hondo) cluster.
(9.43 MB TIF)
