# Supplementary figures and images for: Genome-Wide Association Study of Pancreatic Cancer in Japanese Population
Source: PLoS One. 2010 Jul 29;5(7):e11824. doi: 10.1371/journal.pone.0011824 (PMC2912284; doi:10.1371/journal.pone.0011824)

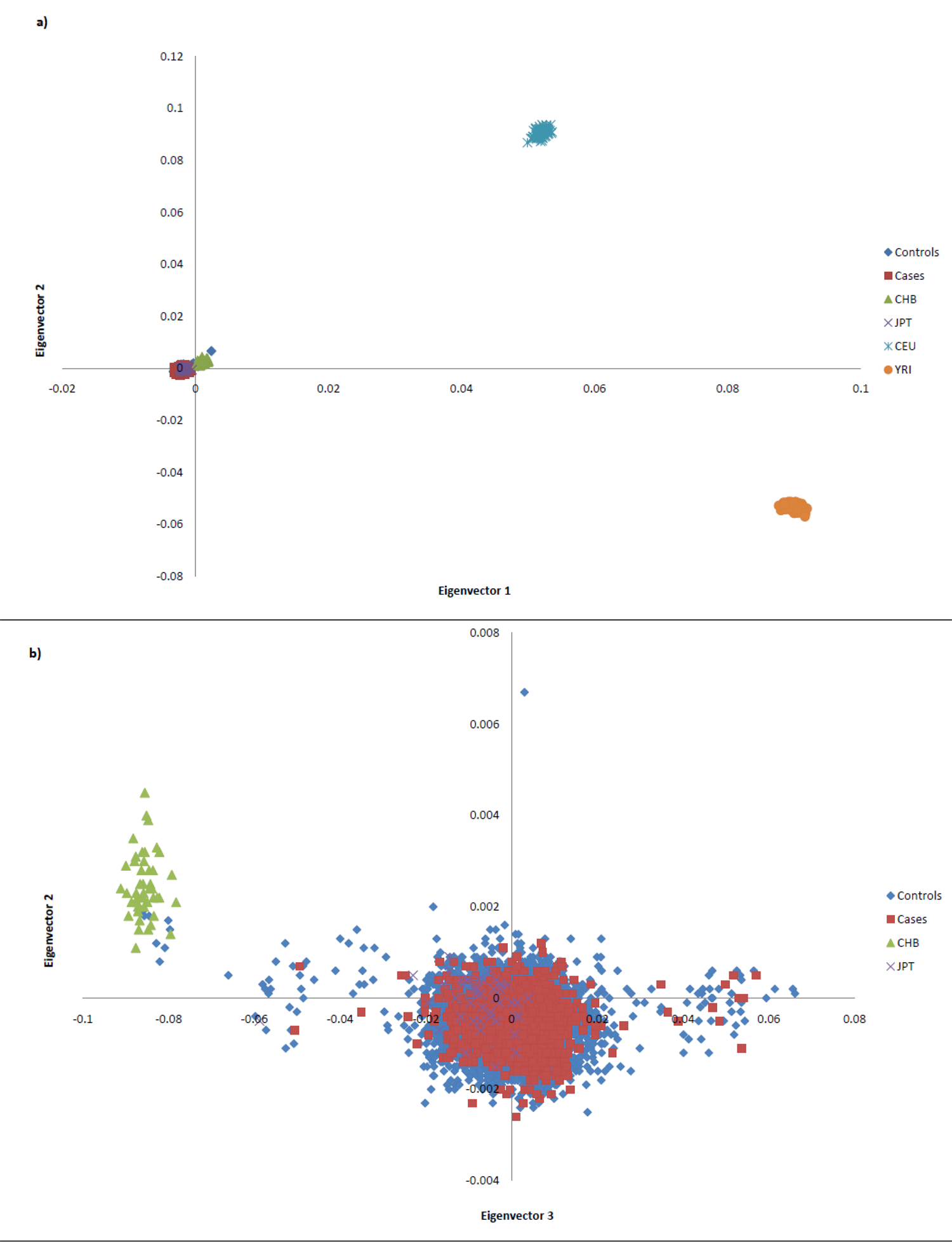

Supplement: Figure S1 — Principal component analysis for GWAS of pancreatic cancer in Japanese population. a) Principal component analysis for GWAS of pancreatic cancer in Japanese population refer to four HapMap population control subjects including CEU indicates Caucasians from Utah; YRI, Nigerians from Yoruba; CHB, Han Chinese from Beijing and JPT, Japanese from Tokyo. b) Principal component analysis of study subjects referred only to Asian populations. We utilized samples from the homogenous case-control (Hondo) cluster. (9.43 MB TIF) [file pone.0011824.s005.tif]
